# From stasis to systematics: deciphering the pathophysiology of secondary lymphedema through omics

**DOI:** 10.3389/fimmu.2026.1850725

**Published:** 2026-07-20

**Authors:** Annica R. Stull-Lane, Xizhao Chen, Abraham J. Book, Radomir Kratchmarov, Sarit Pal, Jinyeon Shin, Gopika Ashokan, Geoffrey E. Hespe, Babak J. Mehrara, Raghu P. Kataru

**Affiliations:** 1 Division of Plastic and Reconstructive Surgery, Department of Surgery, Memorial Sloan Kettering Cancer Center, New York, NY, United States; 2 Advanced Computing and Oncology Laboratory, Department of Radiation Oncology, Memorial Sloan Kettering Cancer Center, New York, NY, United States; 3 Division of Allergy and Clinical Immunology, Department of Medicine, Brigham and Women’s Hospital, Harvard Medical School, Boston, MA, United States

**Keywords:** artificial intelligence, genomics, lipidomics, metabolomics, omics technologies, proteomics, secondary lymphedema, transcriptomics

## Abstract

Secondary lymphedema (LE) can ensue after disruption of lymphatic vasculature, which may be caused by infection, surgery, or cancer treatment. Omics technologies can move the field beyond an anatomic description of lymphatic stasis by defining inflammatory, fibrotic, metabolic, lymphatic vascular, and genetic susceptibility programs that shape disease onset and progression. This review summarizes studies that use transcriptomic, proteomic, metabolomic, lipidomic, and emerging genomic or computational approaches in secondary LE. We synthesize how these datasets have identified candidate biomarkers, cell populations, signaling pathways, and therapeutic targets; highlight limitations of current platforms, samples, and bioinformatic pipelines; and propose future multi-omics strategies for diagnosis, risk stratification, and treatment development of secondary LE.

## 1 Introduction

Secondary lymphedema (LE), a chronic debilitating disease with no approved pharmacologic treatment, involves injury to lymphatic vessels, fluid stasis, immune activation, and fibroadipose deposition (1, 2). The NIH workshop “Yet to be Charted: Lymphatic System in Health and Disease” identified a growth opportunity in using advanced omics to characterize LE (1). Omics technologies incorporate large datasets and provide a systems-level view of biological processes, yielding information that may aid diagnosis, risk stratification, treatment monitoring, and therapeutic target discovery. Omics tools have been used in lymphatic biology in general; for example, the proteomic composition of lymph fluid has been extensively investigated (3–5). However, fewer studies have applied omics methods specifically to secondary LE. How have transcriptomics, proteomics, metabolomics, lipidomics, and emerging genomic and computational approaches advanced understanding of secondary LE? This review summarizes the current literature (**Table 1**), emphasizes functional interpretation and translational relevance, highlights methodological limitations, and proposes future multi-omics strategies for the field.

**Table 1:** Summary of omics-related studies on secondary LE.

Study	Related pathology	Model	Subjects	Sample type	Technology
Adipose cells and related samples					
Karaman et al. (6)	BCRL	Human	Paired affected and unaffected arms (N = 20); compared to lean and obese cohort without LE	Subcutaneous adipose tissue	RNA-seq, metabolome, lipidome
Zamora et al. (7)	BCRL	Human	Paired normal and LE arm (N = 4-5)	Dermolipectomy tissue sample	RNA-seq, and lipidome
Zhao et al. (8)	Secondary LE (cervical, endometrial, ovarian cancers)	Human	Secondary LE (N = 19) and healthy individuals (N = 18)	Subcutaneous fatty tissue	RNA-seq
Chen et al. (83)	Cancer-related (cervical or penile) secondary LE	Human	Normal control (N = 3) and LE (N = 5)	Subcutaneous adipose tissue	RNA-seq
Park et al. (10)	Upper extremity LE	Human	Controls (N = 5) and LE patients (N = 5)	Subcutaneous adipose tissue	RNA-seq
	Hindlimb LE model	Mouse	Untreated LE (N = 4), VLNT-treated (N = 4), VLNT with ABTAA (N = 4), all 8 weeks post-surgery	Mouse hindlimb soft tissue	RNA-seq
Westcott et al. (12)	LE (secondary>primary), both arm and leg	Human	Paired unaffected control and LE limbs (N = 7)	Subcutaneous adipose tissue	Single-nucleus RNA sequencing
Liu et al. (13)	Cervical cancer-related thigh LE	Human	Thigh liposuction specimens of healthy donors (N = 4) and cervical cancer patients (N = 5)	Stromal vascular fraction (all non-adipose cells) of subcutaneous adipose tissue	scRNA-seq transcriptomics
Chen et al. (9)	Cervical cancer-related thigh LE	Human (reanalysis)	Reanalysis of Liu et al. (13): Thigh liposuction specimens of healthy donors (N = 4) and cervical cancer patients (N = 5)	Stromal vascular fraction (all non-adipose cells) of subcutaneous adipose tissue	scRNA-seq transcriptomics
Levi et al. (14)	BCRL	Human	3 human lipoaspirate of upper extremity from LE 3 control patients undergoing cosmetic liposuction procedures	Adipose-derived stem cells from subcutaneous adipose tissue	Transcriptional profiling with microfluidics chip of sorted single adipose-derived stem cells
Xiang et al. (15)	Gynecologic cancer	Human	Thigh liposuction from patients with gynecologic cancer (N = 10), as compared to normal upper abdomen of same patients	Adipose-derived mesenchymal stem cells identified from the stromal vascular fraction of adipose tissue	RNA-seq
Andersen et al. (16)	BCRL	Human	Abdomen or thigh liposuction then injected donor adipose-derived regenerative cells + lipotransfer (N = 39) vs Lactated Ringer’s solution + lipotransfer (N = 41) to assess BCRL treatment; scRNA-seq compared responders (N = 5) vs nonresponders (N = 5)	Donor adipose-derived regenerative cells	scRNA-seq transcriptomics
Hwang et al. (17)	Creeping fat in Crohn’s disease patients, cervical cancer-related thigh LE	Human	Paired mesenteric adipose tissue attached to inflamed or uninflamed ileum from Crohn’s disease patients (N = 3) and ulcerative colitis patients (N = 2) undergoing bowel resection compared with LE patients (N = 5) from Liu et al. (13).	Adipose tissue	scRNA-seq transcriptomics
Li et al. (19)	Erysipelas and cancer-associated LE	Human	Non-erysipelas (N = 6) vs Erysipelas (N = 6)	Adipose tissue samples from liposuction (arm or leg)	RNA-seq
Dong et al. (20)	Cancer-associated secondary LE	Human	Normal (N = 10) and cancer-associated secondary LE (N = 40; 10 breast cancer, 30 gynecologic cancers) limb tissue	Adipose tissue	RNA-seq
	Cervical cancer-related thigh LE	Human (reanalysis)	Reanalysis of Liu et al. (13): Thigh liposuction specimens of healthy donors (N = 4) and cervical cancer patients (N = 5)	Stromal vascular fraction (all non-adipose cells) of subcutaneous adipose tissue	scRNA-seq
Huang et al. (23)	Gynecologic cancer	Human (reanalysis)	Reanalysis of Xiang et al. (15): Thigh liposuction from patients with gynecologic cancer (N = 10), as compared to normal upper abdomen of same patients	Adipose-derived mesenchymal stem cells identified from the stromal vascular fraction of adipose tissue	RNA-seq
	Upper extremity LE	Human (reanalysis)	Reanalysis of Park et al. (10): Controls (N = 5) and LE patients (N = 5)	Subcutaneous adipose tissue	RNA-seq
	Cancer-related (cervical or penile) secondary LE	Human (reanalysis)	Reanalysis of Chen et al. (83): Normal control (N = 3) and LE (N = 5)	Subcutaneous adipose tissue	RNA-seq
	Cervical cancer-related thigh LE	Human (reanalysis)	Reanalysis of Liu et al. (13): Thigh liposuction specimens of healthy donors (N = 4) and cervical cancer patients (N = 5)	Stromal vascular fraction (all non-adipose cells) of subcutaneous adipose tissue	RNA-seq
Chen et al. (26)	Upper extremity LE	Human (reanalysis)	Reanalysis of Park et al. (10): Controls (N = 5) and LE patients (N = 5)	Subcutaneous adipose tissue	RNA-seq
	Hypertrophic scar	Human (reanalysis)	Reanalysis of GSE178411 using select uninjured skin normal controls (N = 24) and hypertrophic scar samples (N = 28)	Skin	RNA-seq
	BCRL	Human (reanalysis)	Reanalysis of Karaman et al. (6): Select paired affected and unaffected arms (N = 10), stratified by short-term (<3 years) and long-term (>14 years) disease	Subcutaneous adipose tissue	RNA-seq
	Cervical cancer-related thigh LE	Human (reanalysis)	Reanalysis of Liu et al. (13): Thigh liposuction specimens of healthy donors (N = 4) and cervical cancer patients (N = 5)	Stromal vascular fraction (all non-adipose cells) of subcutaneous adipose tissue	RNA-seq
Sedger et al. (78)	LE (secondary>primary), arm or leg	Human	LE adipose tissue donor (N = 15); normal adipose tissue donor (N = 7); normal blood donor (N = 11)	Liposuction adipose tissue	Fatty acid analysis, lipidomics
Skin samples					
Lin et al. (30)	LE	Human	Paired LE (N = 63) and normal (N = 27) skin samples from same individuals	Skin punch biopsy	Whole genome microarray
Park et al. (31)	BCRL	Human	Paired unaffected control and LE limbs (N = 4)	Skin biopsy	RNA-seq
Fu & Liu (32)	Tail excision LE model	Mouse	Reanalysis of Gousopoulos et al. (46): Control (normal tail tissue, N = 5), acute LE (2 weeks TLE, N = 5), chronic LE (6 weeks TLE, N = 4)	Tail skin	RNA-seq
Tabibiazar et al. (34)	Tail excision LE model	Mouse	3 groups: normal control, 2-week surgical sham, and 2-week TLE LE; each group with 3 biological replicates, each replicate from pooled samples of 3 mice	Tail skin	Microarray
Qiu et al. (38)	Tail excision LE model	Mouse	Reanalysis of Tabibiazar et al. (34): 2 groups: normal control, 2-week TLE LE; each group with 3 biological replicates, each replicate from pooled samples of 3 mice	Tail skin	Microarray
Shukla et al. (44)	Radiotherapy	In vitro human cell culture	Radiotherapy (10Gy) vs no radiotherapy (0Gy) (vs 2Gyx5 for LECs and ADSCs)	Cell culture of skin and subcutaneous tissues (normal dermal fibroblasts, normal human epidermal keratinocytes, human placental pericytes, microvascular blood endothelial cells, LECs, ADSCs)	RNA-seq
Wolf et al. (45)	Tail excision LE model	Mouse	Control (2 weeks TLE, N = 10) and anti-CTLA4 treated (2 weeks TLE, N = 10) female mice	Tail skin	RNA-seq
Gousopoulos et al. (46)	Tail excision LE model	Mouse	Unoperated control (N = 5), 2 weeks TLE (N = 5), and 6 weeks TLE (N = 5) mice	Tail skin	RNA-seq
Collecting lymphatic vessels, lmc, and lec samples					
Zawieja et al. (49)	LE	Mouse	IALVs from male (N = 5) and female (N = 5) mice	Collecting lymphatic vessels	scRNA-seq transcriptomics
Lei et al. (50)	LE	Mouse	IALVs from male and female C57BL/6J mice across the lifespan	Collecting lymphatic vessels, focus on LMCs	scRNA-seq transcriptomics
Arroyo-Ataz et al. (51)	LE	Mouse	Lymphatic vessels and adjacent veins in mouse hindlimb (N = 6) and inguinal-axillary region (6 IALVs from 3 mice)	Focus on LMCs and blood vascular SMCs	scRNA-seq transcriptomics
Schulz et al. (52)	Secondary LE	Mouse	Lymphatic vessels and surrounding adipose and connective tissue pooled from 4 different anatomic regions for male (N = 4) and female (N = 4) C57BL/6J mice	Collecting lymphatic vessels and surrounding tissues	scRNA-seq transcriptomics
Becker et al. (55)	LEC in secondary LE	In vitro human cell culture	3 foreskin-derived LEC isolates in normoxia (21% O2) vs hypoxia (1% O2)	LECs	RNA-seq
Johnson et al. (56)	Secondary LE (lymphatic filariasis)	In vitro human cell culture	hLECs transfected with mimics of bma-miR-5864, bma-miR-86, or a negative control	LECs	RNA-seq
Nelson et al. (76)	Secondary LE	Sheep	Control vs remodeled post-lymphatic injury	LMCs	Proteomics
Blood/serum samples					
Scalise et al. (58)	Various secondary LE and one primary; may be upper extremity or lower extremity	Human	Lipedema (N = 8), LE (N = 8), obesity I (N = 4), obesity II (N = 4)	Platelets	RNA-seq
Yang et al. (62)	Secondary LE, unilateral lower limb (gynecologic cancer)	Human	Unilateral LE (N = 25) vs no LE (N = 26); paired samples before and after LVA	Monocytes (CD14+ cells from peripheral blood mononuclear cells)	RNA-seq
Lin et al. (30)	LE	Human	LE (N = 27) and healthy controls (N = 12)	Serum	Protein targets
Meng et al. (70)	Limb LE	Human	Primary LE vs secondary LE vs normal controls	Serum proteins	Proteomics
Yim et al. (71)	BCRL	Human	LE group (N = 25), RISK group no LE (N = 18), Control group no LE (N = 17)	Serum proteins Serum lipid levels	Proteomics, lipid levels
Yang et al. (72)	Unilateral lower limb LE	Human	Pre-LVA and post-LVA serum samples; 16 patients total; combinations of pooled samples for analysis	Serum proteins	Proteomics
Lee et al. (77)	BCRL	Human	Selenium-treated (N = 15) at 2 weeks, or the placebo-controlled (N = 14) groups at 2 weeks	Serum	Metabolomics
Other tissue samples					
Mohan et al. (39)	Tail excision LE model	Mouse	Control TNTSham given pCMV6 expression vector backbone alone (N = 6) and TNTProx1 given pCMV6-Prox1 (N = 6), both groups treated with 4-week TLE	Tail tissue	RNA-seq
Yuan et al. (43)	Tail excision LE model	Mouse	Unoperated control mice (N = 4) and 12-week TLE mice (N = 4)	Tail tissue	Whole genome microarray
Lee et al. (73)	Secondary LE	Mouse	Sham-operated vs LE-operated	LE-induced right leg tissue vs normal left leg tissue	Proteomics
Genomics and genetic risk profiling					
Leppänen et al. (64)	Primary LE genetics; contextual susceptibility for secondary LE	Human	Individuals with suspected primary LE screened by WES	Germline DNA	Whole-exome sequencing
Agollah et al. (65)	Primary and acquired LE within one family	Human	Single family with lymphatic disease	Germline DNA	Whole-exome sequencing
Leung et al. (66); Visser et al. (67); Kapellas et al. (68); Miaskowski et al. (69)	Risk of BCRL and secondary LE	Human	BCRL cohorts and systematic reviews	Germline DNA	Targeted SNP/genetic association analysis

BCRL, breast cancer-related lymphedema; LE, lymphedema; VLNT, vascularized lymph node transfer; ABTAA, angiopoietin 2-binding and TIE2-activating antibody; scRNA-seq, single-cell RNA-seq; TLE, tail lymphatic excision; ADSC, adipose-derived stem cells; LEC, lymphatic endothelial cell; hLEC, human lymphatic endothelial cell; LMC, lymphatic muscle cell; IALV, inguinal-axillary lymphatic vessel; SMC, smooth muscle cell; LVA, lymphaticovenous anastomosis; TNT, tissue nanotransfection; WES, whole-exome sequencing; SNP, single-nucleotide polymorphism.

## 2 Transcriptomics

**Figure 1:**
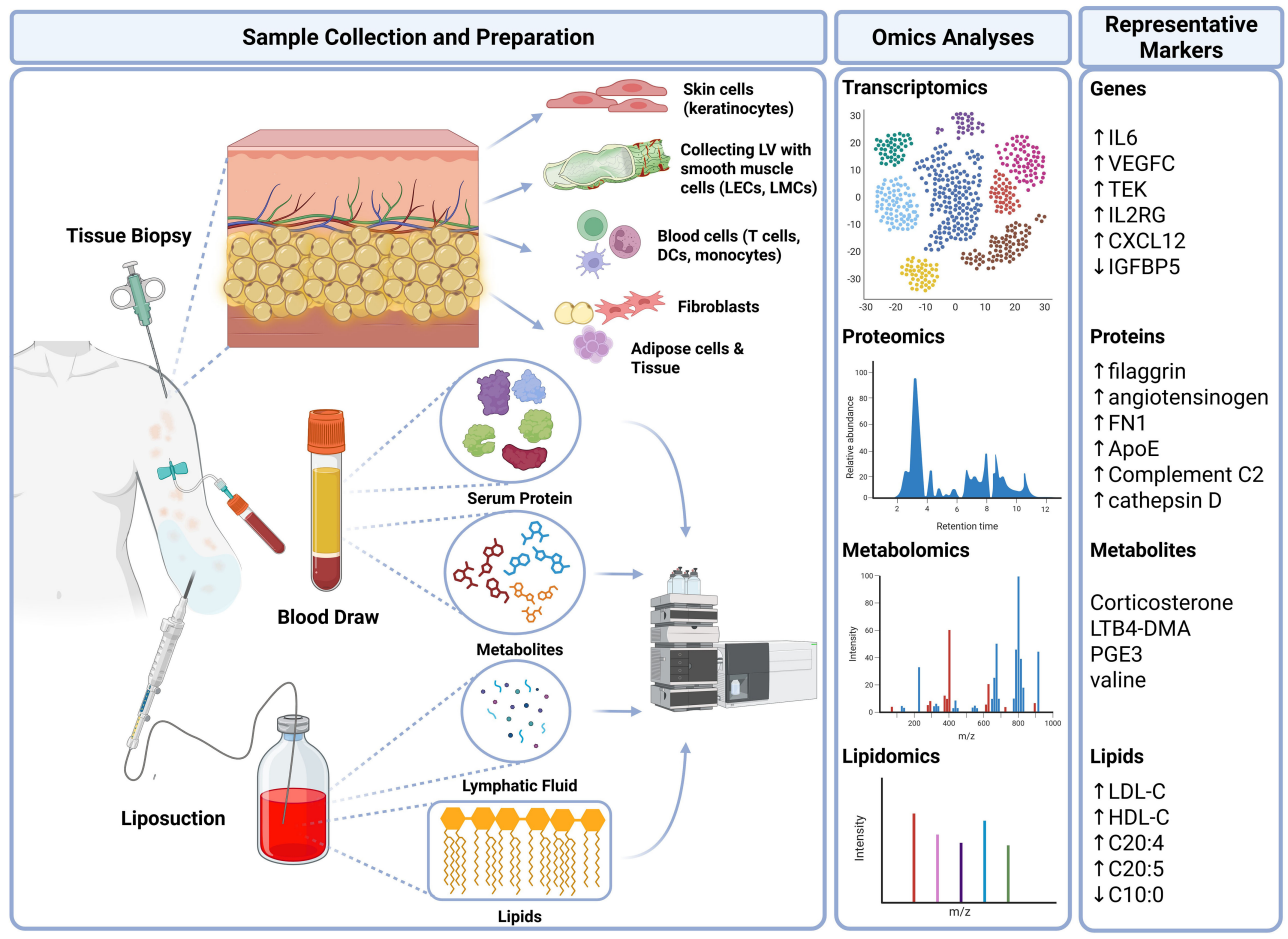
Omics-based sample acquisition, analytical platforms, and candidate molecular markers in secondary lymphedema. Secondary lymphedema can be studied using complementary biospecimens collected by tissue biopsy, blood draw, and liposuction. Tissue sampling enables analysis of skin cells (keratinocytes), collecting lymphatic vessels and associated lymphatic endothelial cells (LECs) and lymphatic muscle cells (LMCs), blood cells (T cells, dendritic cells, monocytes etc.), fibroblasts, and adipose tissue. Blood-based sampling provides serum proteins and circulating metabolites, whereas liposuction-derived material can be used to profile lymphatic fluid-associated metabolites and adipose lipid composition. These specimens support multiple omics approaches, including transcriptomics, proteomics, metabolomics, and lipidomics. Representative markers identified across published studies include inflammatory, lymphangiogenic, immune, and fibrotic genes such as *IL6*, *VEGFC*, *TEK*, *IL2RG*, *CXCL12*, and *IGFBP5*; serum or tissue-associated proteins such as filaggrin, angiotensinogen, fibronectin 1 (FN1), apolipoprotein E (ApoE), complement C2, and cathepsin D; metabolites including corticosterone, LTB4-DMA, PGE3, and valine; and lipid alterations including LDL-C, HDL-C, arachidonic acid (C20:4), eicosapentaenoic acid (C20:5), and decanoic acid/capric acid (C10:0). Because circulating and tissue metabolites are highly influenced by diet, medications, cancer treatment history, body mass index, renal/hepatic function, infection, inflammation, and timing of sample collection, metabolomic findings should be interpreted within the relevant clinical context rather than as disease-specific markers in isolation. Together, these platforms capture the inflammatory, vascular, immune, metabolic, and fibroadipose remodeling processes that characterize secondary lymphedema and may inform biomarker development and therapeutic discovery. The illustration was created in BioRender (https://BioRender.com).

Transcriptomics is the study of all RNA transcripts. A variety of biological samples has been assessed in transcriptomic analyses in studies of secondary LE, with adipose tissue, skin, lymphatic vessel cells such as lymphatic muscle cells (LMCs) and lymphatic endothelial cells (LECs), and blood being the most common (**Figure 1**).

### 2.1 Adipose cells and related samples

Overall, adipose tissue is the most common sample type assessed with transcriptomic techniques, including RNA sequencing (RNA-seq), single-nucleus RNA sequencing (sNuc-seq), and single-cell RNA sequencing (scRNA-seq). LEC dysregulation, chronic inflammation, and fibroadipose deposition are key components of LE progression. Several studies have looked at paired normal and lymphedematous arms in breast cancer-related LE (BCRL). For example, Karaman et al. looked at paired affected and unaffected arms (N = 20) with RNA-seq and found that genes related to the inflammatory response, cell chemotaxis, and angiogenesis were upregulated in the LE arm, whereas genes related to epidermal differentiation, cell-cell junction organization, and water homeostasis were downregulated in the LE arm (6). Zamora et al. compared dermolipectomy tissue samples between paired normal and lymphedematous arms with RNA-seq (7). Upregulated genes in LE included pentraxin 3 (*PTX3*), amphiregulin (*AREG*), interleukin-6 (*IL6*), vascular endothelial growth factor C (*VEGFC*), fms-related tyrosine kinase 4 (*FLT4*), tenascin C (*TNC*), and multiple A disintegrin and metalloproteinase with thrombospondin motifs (*ADAMTS*) genes. Zhao et al. used RNA-seq to investigate differences between subcutaneous fatty tissue from patients with secondary LE versus healthy controls (8). The authors focused on the secretory phospholipase A2 (sPLA2) family of lipolytic enzymes. They found that within the sPLA2 family-related genes, *PLA2G2A* and *PLA2G5* were upregulated in LE. The authors go on to investigate sPLA2-producing macrophages as contributing to LEC dysfunction in the development of LE. Chen et al. utilized RNA-seq of subcutaneous adipose tissue from LE and control patients and found 2025 upregulated and 1285 downregulated genes in LE tissue, gene set enrichment analysis (GSEA) demonstrated that peroxisome proliferator-activated receptor gamma (*PPARG*) signaling was significantly downregulated in LE tissue (9). This finding led the authors to investigate enhancer of zeste homolog 2 (*EZH2*) as an epigenetic regulator of *PPARG*. They discovered that therapeutic inhibition of the EZH2-PPARγ axis reduced fibroadipose tissue in secondary LE.

In addition, Park et al. analyzed subcutaneous adipose from upper extremity LE patients in comparison to control with RNA-seq, identifying >170 differentially expressed genes (10). GSEA demonstrated significantly decreased enrichment scores for multiple lymphatic vascular-related pathways in LE patients as compared with controls. These pathways included endothelial cell activation, lymphangiogenesis, and TIE2 signaling. Based on these findings, the authors went on to investigate activation of TIE2 as decreasing LE in a mouse model. Encoded by the *TEK* gene, TIE2 is a receptor in lymphatic and vascular endothelial cells that promotes lymphatic and vascular integrity and reduces permeability through the angiopoietin-TIE2 pathway (11). Park et al. investigated the role of TIE2 signaling in LE in the mouse hindlimb LE model, assessing both the surgical intervention vascularized lymph node transfer (VLNT) and treatment with the angiopoietin 2-binding and TIE2-activating antibody (ABTAA) (10). The authors utilized RNA-seq to assess hindlimb soft tissue, comparing 3 groups: untreated LE, VLNT-treated, and VLNT with ABTAA. Co-therapy yielded the best outcomes with decreased limb volume and decreased lymphatic leakage. Differential gene analysis informed pathway enrichment, and the pathways with a significantly higher enrichment score in VLNT with ABTAA in comparison to LE only or VLNT alone included TIE2 signaling and vascular wound healing. The authors suggested that a permeable inflammatory microenvironment from lymphatic injury leads to TIE2 downregulation, resulting in compromised lymphatic integrity. Treatment with ABTAA, however, may stabilize endothelial junctions, decrease leakage, and reduce inflammation and fibrosis.

Westcott et al. performed sNuc-seq on subcutaneous adipose tissue from paired affected and unaffected limbs of LE patients (12). sNuc-seq, also known as snRNA-seq, is a transcriptomic method that sequences RNA from inside cell nuclei instead of whole cells. General cell populations identified included LECs, blood endothelial cells, T cells, natural killer (NK) cells, dendritic cells, macrophages, monocytes, smooth muscle cells, pericytes, adipocytes, and adipose stem and progenitor cells (ASPCs). Six subpopulations of adipocytes were described, and acute phase serum amyloid A (SAA)^+^ adipocytes were at a significantly higher proportion of adipocytes in LE as compared to control. SAA^+^ adipocytes were found to be the most differentiated adipocyte subpopulation. Of the six subpopulations of ASPCs identified, the subpopulation characterized by increased expression of VEGFC and the surface receptor glutamate receptor 1 (GRIA1) was found to be greatly increased in LE. The authors bioinformatically assessed cell-cell communication networks and identified increased VEGF network communication from ASPCs to LECs. They ultimately concluded that ASPCs likely contribute to both adipocyte differentiation and lymphatic capillary remodeling.

Adipose tissue as a biological sample is sometimes processed so that the stromal vascular fraction (all non-adipose cells) of the subcutaneous adipose tissue is assessed. Within this fraction, adipose-derived mesenchymal stem cells have also been identified. Both Liu et al. and Chen et al. analyzed a scRNA-seq transcriptome dataset from patients with cervical cancer-related thigh LE (9, 13). Liu et al. identified 21 cell clusters, including populations of adipose-derived stromal cells (ASCs), macrophages, NK cells, NKT cells, dendritic cells, mast cells, pericytes, B cells, T cells, and endothelial cells. The authors noted that cells with expanded populations in LE included CD4^+^ T cells, NK cells, NKT cells, and c3-ASC, one of the ASC populations. In a reanalysis of the same dataset, Chen et al. identified PDGFRA-expressing cells that had increased expression of fibrogenic metalloproteinases (ADAMTSL1) in LE.

Several studies have investigated transcriptomics of adipose-derived stem cells. For example, Levi et al. did transcriptional profiling of adipose-derived stem cells from subcutaneous adipose tissue of 3 patients with upper extremity LE and 3 control patients undergoing cosmetic liposuction procedures (14). Genes with significantly different expression between groups included *CDKN1A*, *NFKB1*, *FOXO3*, *THY1*, *LPL*, *MYC*, *CD44*, and *HIF1A*. A separate study used RNA-seq to investigate adipose-derived mesenchymal stem cells from liposuction samples of patients with gynecologic cancer as compared to autologous abdominal samples from the same patients (15). Upregulated genes included cell cycle regulator genes, and downregulated genes included several cytokines and chemokines. Yet another study utilized donor adipose-derived regenerative cell injections with lipotransfer as compared to placebo with scRNA-seq (16). Interestingly, no differences were found.

Some studies have compared LE to other conditions, such as Crohn’s disease, or investigated conditions that exacerbate LE, such as infections. Both Crohn’s disease and LE involve pathology of adipose tissue, inflammation, and fibrosis. Hwang et al. looked at pro-fibrotic preadipocytes (PACs) in both LE and creeping fat from patients with Crohn’s disease and found in cell-cell interactions that both pathologies showed increased outgoing signals from PACs that included *LIGHT*, *CCL*, *SEMA3*, and *ANNEXIN* (17). In both pathologies, PACs featured upregulated *PTX3* expression, which is important in inflammation. In fact, PTX3 is found in the extracellular matrix of specialized lymphatic vessels, playing an important role in the function of the lymphatic system during inflammation (18). Li et al. used RNA-seq to look at adipose tissue samples from liposuction cases in patients with cancer-associated LE with and without erysipelas, a bacterial infection that can exacerbate LE (19). The authors found that prior erysipelas could lead to worse LE through increased expression of genes relating to metabolic pathways (insulin and glucagon), inflammation, and tissue remodeling. In particular, the TGF-β pathway gene *TGFB2* and several MAPK-related genes were confirmed with reverse transcription quantitative PCR (RT-qPCR).

Dong et al. utilized machine learning algorithms on RNA-seq data from control and cancer-associated secondary LE patient adipose tissue to identify molecular markers of disease and develop predictive models (20). The identified biomarkers included interleukin-2 receptor gamma chain (*IL2RG*), homeobox D10 (*HOXD10*), and tetraspanin1 (*TSPAN1*), all of which were subsequently validated as being upregulated in LE with RT-qPCR. Further, they used scRNA-seq data from Liu et al. to explore interactions between immune cell subpopulations and genes, and the authors found that the three identified genes had relatively higher activity in T cell clusters (13). IL2RG is the common gamma chain in the receptor complex for multiple interleukins, including IL-4 (21), an important cytokine involved in differentiation of CD4^+^ T cells to Th2 cells in LE pathogenesis. HOXD10 is a transcription factor of the homeobox gene family that has been studied in relation to LEC function (22). TSPAN1 is a member of the transmembrane 4 superfamily; in the context of LE, it may be related to Th17 cell differentiation and/or the fibrosis phenotype mediated by epithelial-to-mesenchymal transition (20).

Huang et al. utilized a multi-omics approach with transcriptomic datasets from adipose tissue to computationally characterize the immune microenvironment of secondary LE, focusing on CD4^+^ T cells (23). In combining RNA-seq data from three LE studies and correcting for batch effect, they showed significant upregulation of chemokine C-X-C motif chemokine ligand 12 (*CXCL12*) in the secondary LE group. The authors also reanalyzed available scRNA-seq data and highlighted that the proportion of activated CD4^+^ T cells was increased in LE (41.9%) in comparison to control (17.7%). Further bioinformatic analysis revealed that CD4^+^ T cells were key receivers and integrators of signals from the stromal microenvironment, particularly from fibroblasts through the CXCL12-CXCR4 signaling pathway. The authors used RT-qPCR to validate the upregulation of *CXCL12* in a mouse model of LE. Lastly, they used an *in silico* virtual knockout of *CXCL12* with the scRNA-seq transcriptional data to identify perturbations in gene expression. Previous studies have characterized CXCL12 as important in lymphangiogenesis (24), although can also contribute to T cell chemotaxis and activation (25).

Chen et al. utilized multiple pre-existing transcriptomic datasets to employ an integrated multi-omics approach in identifying novel genes and pathways predictive of LE-related fibrosis (26). The authors employed cancer-related LE and hypertrophic scar RNA-seq datasets to identify 154 fibrosis-related genes. The authors created a protein-protein interaction network and used the top five hub genes in exploratory machine learning analysis with random forest and least absolute shrinkage and selection operator LASSO regression. The most consistent fibrosis-associated genes were *ASPN*, *FMOD*, and *MFAP5*. The authors incorporated analysis of an additional RNA-seq dataset and determined that *ASPN* was most consistently represented in long-term LE cases. ASPN is a protein involved in extracellular matrix remodeling and TGF-β signaling (27), and studies have described its expression by fibroblasts (28). Next, Chen et al. conferred with an available scRNA-seq dataset and identified adipose-derived mesenchymal stem cells as the predominant *ASPN*-expressing population (26). *ASPN* expression correlated with disease duration and fibrosis severity, suggesting that it may be a useful biomarker for fibrotic progression in LE.

Together, these transcriptomic studies across whole adipose tissue, stromal vascular fractions, and adipose-derived stem cells reveal that LE is characterized by widespread inflammatory activation, fibroadipose remodeling, immune cell expansion, and altered metabolic signaling—processes that are further shaped by comorbid conditions and infections—highlighting adipose tissue as a central and dynamic driver of LE progression.

### 2.2 Cells from human skin and/or mouse tail samples

While typically thought of as a disease of the lymphatics, LE also involves skin pathology, including skin barrier disruption and increased susceptibility to skin infections (29). Transcriptomics of skin tissue has been assessed in secondary LE, including human and mouse samples. Lin et al. looked at skin punch biopsies from LE (N = 63) and normal (N = 27) skin samples using whole genome microarray analysis (30). The authors found that certain pathways were significant in analysis using GeneSpring software, including *TGFBR*, *EGRF1*, and *IL6*. Significantly enriched Kyoto Encyclopedia of Genes and Genomes (KEGG) pathways involved multiple biological processes, including immune regulation, metabolic and endocrine signaling, cell survival and tissue remodeling, and angiogenic signaling. Park et al. utilized RNA-seq to assess paired skin biopsy samples from the lymphedematous limb and contralateral limb of BCRL patients (31). The authors found that the LE limb samples had increased expression of fibrotic genes, including TGF-βs, collagens, vimentin, platelet-derived growth factor β, and matrix metalloproteinases. Taken together, these significant pathways highlight important features of chronic LE, including fibrosis, inflammation, and alterations in cutaneous adipose biology.

A more recent study looked at the mouse tail lymphatic excision LE model and did RNA-seq analysis of skin samples (32). The authors found that the following markers were positively correlated with CD4^+^ T cells: *BATF*, *CCR6*, *IL12RB1*, *FOXP3*, and *CD74*. *IL1B* and *IRF4* were negatively related to CD4^+^ T cells. CD4^+^ T cells have been implicated as a key cell type; they are activated in regional lymph nodes and migrate to the skin to contribute to LE disease pathology (33).

Tabibiazar et al. looked at the transcriptional profile of mouse tail skin at 2 weeks post-surgery versus controls (34). The authors found no statistically significant differences between normal control mice and surgical control mice, but they did identify 429 upregulated and 183 downregulated genes in the LE tail skin in comparison to control. Changes in gene expression were related to multiple processes, including acute inflammation, wound healing and fibrosis, stress response, angiogenesis, cytoskeletal organization, Wnt pathway activation, and adipogenesis. The authors used RT-qPCR to confirm several differentially expressed genes. Genes found to be upregulated at 2 weeks post-surgery included calgranulin A (S100a8), calgranulin B (S100a9), matrix metalloproteinase 3 (Mmp3), and Mmp14. S100A8/S100A9 make up the calprotectin protein complex, which is often produced by neutrophils and macrophages during inflammation, contributing to leukocyte recruitment and cytokine secretion (35). While not extensively studied as related to LE progression, it has been reported as related to skin barrier dysfunction (35). Matrix metalloproteinases, components of the extracellular matrix, can degrade structural proteins, allowing for adipose tissue expansion (36). For instance, one study on Mmp14 demonstrated its role in directly cleaving LYVE-1 on lymphatic endothelial cells, inhibiting lymphangiogenesis (37).

Qiu et al. did a reanalysis of the normal control group and LE group microarray data from Tabibiazar et al. (34) and found 307 upregulated genes and 174 downregulated genes in LE (38). The authors applied the maximal clique central method to identify the top 10 hub genes: Fc epsilon receptor Ig (*FCER1G*), CD53 molecule, neutrophil cytosolic factor 4 (*NCF4*), Rac family small GTPase 2 (*RAC2*), lymphocyte antigen 86 (*LY86*), lysosomal protein transmembrane 5 (*LAPTM5*), interferon regulatory factor 8 (*IRF8*), neutrophil cytosolic factor 2 (*NCF2*), allograft inflammatory factor 1 (*AIF1*), and coronin 1A (*CORO1A*). Then the authors did a drug-gene interaction analysis and identified the *ALOX5AP* gene as a possible therapeutic target, since fiboflapon is a known small-molecule inhibitor of the protein that *ALOX5AP* encodes for, 5-lipoxygenase activating protein (FLAP).

Mohan et al. used RNA-seq to study the effect of Prox1 delivered through tissue nanotransfection (TNT) technology in the murine tail LE model (39). The authors compared expression profiles of tail tissue in control (TNTSham) and treated (TNTProx1) animals 4 weeks post-surgery. The TNTProx1 animals showed a reduced inflammatory response in comparison to controls, with the following genes yielding lower transcript levels as confirmed with RT-qPCR: *CCL1*, *CCR4*, *CCL27*, and *TAC1*. In LE progression, skin keratinocytes will express CCL27, which facilitates migration of activated CD4^+^ T cells expressing skin homing receptors like CCR4 (40). CCL1 is a chemokine that interacts with CCR8, a receptor expressed on Th2 cells and T regulatory (Treg) cells, both of which play a role in LE (41). Tac1 encodes for substance P, which is a key neuropeptide involved in pain signaling (42). Prox1 is a master regulator of lymphangiogenesis and thus the authors concluded that therapy with Prox1 likely prevented development of chronic lymphedematous skin changes through increased lymphatic density with faster lymphatic fluid drainage (39).

Yuan et al. also looked at transcriptional changes in the TLE mouse model (43). The authors focused on *IGFBP5* as most strongly downregulated in LE tail tissue, a finding confirmed by RT-qPCR. Since IGFBP5 can bind IGF1 and prevent it from activating the IGF1 receptor (IGF1R), the authors went on to study the therapeutic role of linsitinib, a small-molecule inhibitor of the tyrosine kinase activity of IGF1R. Interestingly, they reported promising results of linsitinib therapy reducing the pathology of chronic LE in the mouse model.

Patients with secondary LE have received other treatments for their cancer, such as radiotherapy. Shukla et al. used RNA-seq to assess *in vitro* cell cultures of human skin cells, including normal dermal fibroblasts and normal human epidermal keratinocytes, with and without radiotherapy treatment (44). The authors found that altered markers for all cell types included *ATF3*, *SELE*, *CDKN1A*, *GDF15*, and *MDM2*, indicating that cell cycle regulation and DNA damage response and repair mechanisms had been affected.

In addition to radiotherapy, immunotherapy may also reprogram the LE microenvironment, impacting disease progression. Both Wolf et al. (45) and Gousopoulos et al. (46) assess transcriptomics changes in the mouse tail model of LE in the context of different treatments. Wolf et al. applied bulk RNA-seq to lymphedematous tail skin from anti-CTLA4 treated and control mice and identified 846 differentially expressed genes (45). GSEA showed downregulated keratinization in the anti-CTLA4 treatment group, suggesting less dermal thickening and an improved LE outcome. The cellular responses to interferon beta and gamma pathways were upregulated in anti-CTLA4 treated animals. Furthermore, molecular processes downregulated in the anti-CTLA4 therapy group included fibrosis, epithelium degeneration, and edema, all suggesting that the treatment improves LE. Overall, the authors found that anti-CTLA4 treatment protected against LE development in patients with melanoma and in a mouse LE model, and that this correlated with systemic expansion of Treg cells.

Gousopoulos et al. applied bulk RNA-seq to mouse tail samples at 0, 2, and 6 weeks after LE surgery (46). The authors found that T cell-related networks were upregulated at 2 and 6 weeks, especially genes associated with Tregs. This finding led the authors to investigate Tregs in the LE context, discovering that Treg depletion in the mouse model exacerbated LE. They found that expansion of Tregs using IL-2/anti-IL-2 mAb complexes significantly reduced LE development, and that adoptive transfer of Tregs at 1 week after LE surgery improved LE outcome in the mouse model. Taken together, these experiments offer potential immunotherapeutic strategies with Tregs directly or antibody-driven expansion of Tregs (47).

Skin samples involve a heterogeneous population of cells, and although RNA-seq is important in elucidating global changes in gene expression, scRNA-seq approaches would add more robust data to these queries on LE pathology. Together, these transcriptomic studies demonstrate that LE-related skin pathology arises from coordinated disruptions in inflammatory, immune, and tissue-remodeling pathways—as well as radiotherapy-induced cellular stress—underscoring the complex molecular environment that drives chronic skin dysfunction in secondary LE.

### 2.3 Collecting lymphatic vessels, LMC, and LEC samples

In LE, the collecting lymphatic vessels typically display compromised contractile activity that is weak and irregular (48). Several studies have applied transcriptomic methodologies to investigate cells related to lymphatic vessels, such as LMCs and LECs. Zawieja et al. utilized scRNA-seq of mouse inguinal-axillary lymphatic vessels (IALVs) to report on multiple cell types including LECs, LMCs, adventitial cells, and immune cells (49). They identify ten distinct LEC subclusters and 4 different LMC subclusters. The authors go on to demonstrate that LMCs are intrinsic pacemakers, suggesting a potential therapeutic target to rescue lymphatic pacemaking in LE patients. In a separate study, Lei et al. applied scRNA-seq to characterize LMCs from collecting lymphatic vessels and found that a pro-inflammatory microenvironment suppressed the contractile apparatus of LMCs and lymphatic vessel pumping in advanced-age mice (50). In addition, Arroyo-Ataz et al. applied scRNA-seq to characterize the transcriptomes of LMCs and smooth muscle cells (SMCs) from mice and found that venous SMCs were more similar to LMCs than arteriole SMCs (51). They also found that Wilms tumor gene 1 (WT1)^+^ mesodermal progenitors give rise to a population of LMCs critical for lymphatic vessel contractility. While a clear limitation of these studies is that mouse data is not always translatable to human, taken together, these murine studies help highlight the importance of LMCs contractility in LE and suggest that potential therapeutics could modulate LMC function to improve lymphatic vessel contractility.

Analyses of cell populations from collecting lymphatic vessels may also illuminate important immune cells that regulate lymphatic vessel contractility. For instance, Schulz et al. utilized scRNA-seq to map the cell populations of mouse collecting lymphatic vessels and neighboring tissues (52). The authors identified 29 different cell type clusters that were associated with 17 different cell types. They found that LECs and lymphatic vessel endothelial hyaluronan receptor 1 (LYVE1)^+^ macrophages had high expression of transient receptor potential vanilloid 4 (*TRPV4*) channels. TRPV4 channel activation was shown to impair LEC contractility. Separately, the authors confirmed that LYVE1^+^ TRPV4^+^ macrophages were increased in BCRL human tissue biopsies in comparison to unaffected control arms. Their data suggested that activation of TRPV4-expressing macrophages leads to release of potent vasoconstrictive prostanoids that contribute to strong and sustained constriction of LMCs. This vasospasm impairs the contractility of collecting lymphatic vessels. A limitation to this study was that cells were pooled from multiple anatomic regions, so the results do not allow for deciphering differences based on anatomy. Nonetheless, in conjunction with the previously discussed contractility studies, this study helps highlight the therapeutic potential of regenerating lymphatic vessels after injury, as properly functioning contractility would help prevent the detrimental fluid build-up in LE pathophysiology.

Cell culture LECs from secondary LE under various treatment conditions have been assessed with RNA-seq. At homeostasis, the physiological oxygen level of the lymphatic system can vary based on anatomical location, but lymphatic vessels and organs are typically accustomed to hypoxic conditions (53). Normally, a decrease in oxygen stabilizes HIF-1α and stimulates lymphangiogenesis by LECs (54). However, the persistent hypoxia seen in LE can lead to dysfunctional vessel growth, progression of inflammation, and fibrosclerosis (54). Becker et al. looked at LEC isolates cultured in hypoxia versus normoxia and concluded that hypoxic LECs can alter the extracellular matrix and contribute to fibrosis (55). Of the 16,000 detected genes, 162 were found to be dysregulated in hypoxic conditions. *Elastin*, *VWA1*, and *ADAMTS15* were found to be downregulated, whereas many genes were found to be upregulated, including *FBLN5, FMOD, MMP1, TGFB2, TGFB3*, and *ADAMTS6*. Separately, other researchers utilized RNA-seq to assess how human LECs (hLECs) respond to microRNAs secreted by the parasitic nematode *Brugia malayi* (56). The authors treated hLECs with microRNAs bma-miR-5864 and bma-miR-86 and found 788 and 811 differentially expressed genes, respectively, as compared to negative control. Fibronectin 1 (*FN1*) and the KEGG terms “extracellular matrix-receptor interactions” and “focal adhesion” were significant, leading the authors to discuss how *Brugia malayi* may disrupt LEC integrity, increasing the permeability of the endothelial barrier and thus contributing to secondary LE pathogenesis. These studies included LECs in cell culture, although future transcriptomic studies could assess LECs *ex vivo* isolated from LE patients as compared to healthy controls. Collectively, these studies highlight LEC dysfunction as a key component of secondary LE. They show that LECs undergo substantial transcriptomic reprogramming in response to hypoxia and pathogen-derived microRNAs, leading to extracellular matrix remodeling, impaired barrier integrity, and pro-fibrotic signaling.

### 2.4 Cells from blood samples

Cells collected from human blood samples, including platelets and monocytes, have been assessed with RNA-seq. A 2024 study found that obese patients with LE were more likely to experience venous thromboembolism than in obese patients without LE (57). The same finding was true for obese patients with and without lipedema, which is a chronic condition characterized by an abnormal buildup of adipose tissue. In a follow-up study, Scalise et al. found that, in comparison with platelets from lipedema patients, platelets from LE patients had decreased angiogenesis signals and decreased signals for inhibiting mitosis (58). The transcriptomes from LE and lipedema platelets also differed in pathways relating to protein synthesis and granule exocytosis. These findings demonstrate that although lipedema and LE both involve adipose deposition and increased risk of venous thromboembolism, they differ in pathophysiology. Platelets assist in maintaining lymphatic vessel integrity and may contribute to LE development if dysregulated (59, 60).

Monocytes are the precursors to macrophages, which infiltrate lymphedematous tissues and contribute to lymphedema pathogenesis (36, 61). Yang et al. looked at the expression of dysregulated genes in circulating monocytes of patients with cancer-related LE. Six hub genes were identified: *CCL2, LPL, PDK4, FOXO3, EGR1*, and *DUSP5* (62). All six hub genes have connections to oxidative stress, which occurs in lymphedematous tissue (63). Protein-protein interaction analysis showed five functional modules involving immunity, lipid metabolism, oxidative stress, transcriptional regulators, and tumor suppression. The study creatively looked at gene expression before and after lymphaticovenous anastomosis (LVA) and identified genes that were recovered, including *SPRY1, SLC1A3, ZBTB16*, and *ALOX15B*. Taken together, RNA-seq technology has effectively been applied to analyzing transcriptomics of components of peripheral blood in secondary LE. However, the comparative analysis of the transcriptome of blood cells like granulocytes (neutrophils, eosinophils, basophils) and lymphocytes in LE patients as compared to other patients, and/or before and after treatments of interest, remains understudied.

## 3 Genomics and genetic risk profiling

Although most omics studies of secondary LE have emphasized transcriptomes, proteomes, metabolites, and lipids, genomic approaches are important for understanding why only a subset of patients develop LE after apparently similar lymphatic injury. Whole-exome sequencing (WES) has been applied primarily to hereditary or primary LE rather than to large secondary LE cohorts. For example, Leppänen et al. used WES to study primary LE and reported ANGPT2-associated lymphatic disease (64), while a family-based WES study implicated an INPPL1 variant encoding SHIP2 in a pedigree with both primary and acquired LE (65). These data do not prove that these variants are common causes of secondary LE, but they support a model in which inherited lymphatic vulnerability may modify the response to surgery, radiation, infection, or obesity-related inflammation.

Targeted genetic association studies provide additional, though still incomplete, evidence for susceptibility to breast cancer-related LE (BCRL). Reported variants include inflammatory cytokine genes such as *IL1B, IL4, IL6, IL10, IL13*, and *NFKB2*; lymphangiogenic or vascular genes such as *VEGFC, FLT4/VEGFR3, KDR/VEGFR2, FOXC2, NRP2, HGF*, and *MET*; and immune or junctional genes such as *LCP2, SYK, VCAM1, RORC, GJC2/CX47*, and *GJA4/CX37* (66–69). These findings should be interpreted as risk-modifying rather than deterministic because effect sizes, ancestry-specific associations, cancer-treatment exposures, and phenotype definitions vary across studies. WES is also inherently limited because it interrogates protein-coding regions and can miss regulatory, structural, mitochondrial, and epigenetic mechanisms. Future work should integrate germline risk profiling with longitudinal clinical data and tissue-level omics to distinguish inherited susceptibility from injury-induced molecular remodeling.

## 4 Proteomics

Proteomics is the study of all proteins in a sample. Discoveries can lead to disease-specific biomarkers and inform potential therapies. Both serum and tissue samples have been assessed.

### 4.1 Serum assessments

Serum is the most common sample assessed with protein assays. In 2012, Lin et al. looked at which proteins were significantly higher in LE serum to create a regression model (30). The authors included six proteins in the model: FGFb, IL4, IL10, TNFb, TGFb, and leptin. A receiver operating characteristic curve yielded an area under the curve (AUC) of 0.87 (95% CI: 0.75 to 0.97). The AUC suggests promising discrimination between LE and healthy controls, although the moderately wide confidence interval and lack of reported sensitivity and specificity at an optimal cutoff limit clinical interpretation. The serum samples were also heterogeneous, with both upper- and lower-extremity LE and only approximately half derived from cancer-related LE. Meng et al. studied normal controls versus secondary LE using nano RPLC-MS/MS and identified 37 proteins with increased expression in secondary LE, including filaggrin, complement factor D, angiotensinogen, and complement C2 (70). ELISA showed that cathepsin D was significantly higher in secondary LE serum than in controls, although it did not correlate with the percentage increase in affected-limb volume. Yim et al. assessed a LE group, a RISK group, and a control group and found differences in FN1, apolipoprotein E (ApoE), antithrombin, and complement C4; ELISA confirmed higher FN1 and ApoE in RISK and LE groups than controls (71). Yang et al. compared serum before and after lymphaticovenous anastomosis and found that CAT, PRDX2, CA1, and hemoglobin subunit alpha changed after surgery, with CAT validated by ELISA (72). These studies support the translational potential of serum biomarker panels for early detection, staging, prognosis, and treatment monitoring, but head-to-head validation is needed before any candidate can be considered clinically robust or disease-specific.

### 4.2 Tissue assessments

In characterizing LE, proteomics has been utilized to study lymphedematous protein profiles in animal models. Lee et al. assessed leg tissue from sham-operated versus LE-operated mice, finding 20 functional proteins that were differentially expressed (73). Among the discussed proteins was serum amyloid P-component precursor (SAP), which was confirmed to be increased in LE tissue by western blot. Given its immunomodulatory role, SAP has been investigated in clinical trials for its therapeutic potential in fibrotic disease, such as pulmonary fibrosis (74). No study to date has investigated it as a LE therapy. While Lee et al. looked at LE leg tissue, other researchers have looked at specific cell types in tissue. LMCs are critical for lymphatic contraction, and their dysregulation is present in LE pathophysiology (75). Nelson et al. looked at secondary LE in sheep, comparing proteomics of lymphatic muscle cells collected from control tissue versus tissue that was remodeled post-lymphatic injury (76). Upregulated protein networks within the wounded system included components of cell death, organismal injury, cellular movement, small molecule metabolism, and lipid metabolism. Upregulated pathways in the remodeled muscle cells included oxidative phosphorylation and markers of oxidative stress. While a limitation is that findings from animal studies are not always translatable to the human context, the studies discussed here do demonstrate that lymphatic injury leads to coordinated structural, metabolic, and oxidative changes across affected tissues. These findings highlight conserved biological pathways—such as oxidative stress, lipid metabolism, and tissue remodeling—that underpin the development and persistence of secondary LE.

## 5 Metabolomics

Metabolomics is the comprehensive study of small molecules that reflect dynamic changes in cellular metabolism, redox biology, inflammation, and tissue remodeling. In secondary LE, metabolomics is particularly useful because it captures disease-associated physiology that may not be evident from RNA abundance alone. Its interpretation, however, requires careful attention to diet, fasting status, medications, circadian timing, cancer treatment history, sample handling, and analytical platform, all of which can affect metabolite abundance.

### 5.1 Serum assessments

Lee et al. analyzed serum samples from BCRL patients two weeks after treatment with selenium or placebo and identified 107 differential metabolites (77). The metabolites were categorized into lipids and lipid-like molecules, organic acids and derivatives, organic heterocyclic compounds, and nucleosides and nucleotides. Corticosterone, LTB4-DMA, and PGE3 were elevated in the selenium-treated group. Although this study was designed around a therapeutic intervention rather than untreated disease biology, it illustrates how metabolomics can be used to monitor biochemical response to treatment and to identify candidate pharmacodynamic readouts related to inflammation and lipid mediator pathways.

### 5.2 Tissue assessments

Karaman et al. assessed the metabolome of paired affected and unaffected arm subcutaneous adipose tissue and found reduced valine in LE tissue (6). This finding is not sufficient by itself to define a causal metabolic defect, but it suggests that chronic lymphatic injury may alter amino-acid utilization or tissue metabolic state. Because tissue metabolomes are strongly influenced by local hypoxia, immune infiltration, adipocyte hypertrophy, and systemic nutritional factors, future studies should pair metabolomics with cell-resolved transcriptomics, imaging, and clinical staging.

## 6 Lipidomics

Lipidomics focuses on the composition and bioactive function of lipids, including structural lipids, fatty acids, eicosanoids, and specialized pro-resolving mediators. This platform is functionally distinct from broad metabolomics because lipid mediators can directly regulate vascular permeability, macrophage activation, T-cell function, fibrosis, and resolution of inflammation—processes central to LE progression.

### 6.1 Serum assessments

Yim et al. found that serum LDL-C and HDL-C levels were elevated in the LE group compared with controls (71). These systemic lipid measurements are not disease-specific biomarkers and are affected by metabolic health, medication use, and diet. Nevertheless, they raise the possibility that lipid metabolism, adipose expansion, and lymphatic dysfunction interact bidirectionally in BCRL.

### 6.2 Tissue assessments

Sedger et al. analyzed lipidomic profiles of liposuction aspirates from LE patients and cosmetic-surgery controls (78). They reported a potential global increase in polyunsaturated fatty-acid-containing triacylglycerides, reduced C10:0 (decanoic/capric acid), elevated C20:4 (arachidonic acid), C20:5 (eicosapentaenoic acid), and C22:6 (docosahexaenoic acid) in oil fractions, increased 9,10-methylene hexadecenoic acid, and reduced C24:1 (nervonic acid) in fat fractions. The authors also identified differences in cholesterol esters, phosphatidylcholines, and ceramides. Zamora et al. studied paired dermolipectomy tissue and found that LE arms had reduced arachidonic acid-derived specialized lipid mediators, including 15-lipoxygenase-associated products, 15-hydroxyeicosatetraenoic acid, lipoxin A4, and lipoxin B4 (7). Karaman et al. did not find significant lipidomic differences between paired arms, underscoring that lipidomics may be sensitive to cohort composition, tissue processing, and lifestyle covariates (6). Together, these studies suggest that secondary LE is not only an inflammatory state but also a failure of lipid-mediated resolution, which may be therapeutically actionable if validated in larger cohorts.

## 7 Discussion

### 7.1 Integrated functional synthesis and translational relevance

The collective omics literature indicates that secondary LE is not simply passive fluid accumulation after lymphatic obstruction. Rather, lymphatic injury initiates a self-reinforcing tissue program that includes impaired lymphatic endothelial barrier function, reduced collecting-vessel contractility, chronic immune activation, adipose expansion, extracellular-matrix remodeling, and altered metabolic and lipid-mediator signaling (9, 33, 40, 76, 79–82). Transcriptomic studies of adipose tissue repeatedly identify inflammatory chemotaxis, macrophage and T-cell programs, lymphangiogenic stress signals, and fibroadipose pathways (6, 7, 23–25). These findings are functionally meaningful because they connect the clinical phenotype of swelling and fibrosis to candidate cellular regulators, including LECs, LMCs, LYVE1^+^ macrophages, CD4^+^ T cells, Tregs, adipose stem and progenitor cells, adipocytes, fibroblasts, and PDGFRα^+^ mesenchymal cells.

Several convergent pathways now have translational relevance. The EZH2–PPARγ axis and PDGFRα^+^ fibroadipogenic populations link transcriptomic findings to adipose accumulation and provide a rationale for anti-fibroadipose therapy (83). The TIE2/ANG-TIE2 axis links endothelial integrity and lymphatic leakage to surgical and antibody-based strategies (10). CXCL12–CXCR4 signaling suggests that fibroblast-to-CD4^+^ T-cell communication may help maintain the inflammatory microenvironment (23, 25), whereas ASPN, FMOD, and MFAP5 connect long-standing disease to extracellular matrix remodeling and fibrosis (26). Lipidomic data implicating 15-lipoxygenase-derived mediators and lipoxins suggest that LE may involve impaired resolution of inflammation, not only inflammatory activation (7). These examples demonstrate how omics can move the field from descriptive gene lists toward testable therapeutic hypotheses.

Biomarker discovery is promising but not yet clinically mature. Serum candidates include the Lin et al. six-protein panel (FGFb, IL4, IL10, TNFb, TGFb, and leptin), CTSD, FN1, ApoE, CAT, PRDX2, CA1, and hemoglobin subunit alpha; tissue and blood candidates include IL2RG, HOXD10, TSPAN1, CXCL12, ASPN, FMOD, MFAP5, PPARγ, TIE2, and lipid mediators such as lipoxin A4 and lipoxin B4 (30). No single candidate can yet be considered the best biomarker because the studies differ in sample source, disease stage, control group, cancer type, treatment exposure, validation method, and statistical design. The strongest near-term translational use is therefore likely to be biomarker panels for risk stratification, subclinical detection, treatment monitoring, and patient selection rather than a single diagnostic analyte.

### 7.2 Methodological considerations and bioinformatic interpretation

Each omics platform answers a different biological question and has distinct limitations. Bulk RNA-seq and microarray studies provide tissue-level expression trends but cannot identify which cell type drives a signal without deconvolution or orthogonal validation (84–86). Single cell RNA-seq and single-nucleus RNA-seq improve cell-type and cell-state resolution and have revealed immune, adipose, endothelial, and lymphatic vessel subpopulations, but these technologies are limited by dissociation or nuclear isolation bias, sequencing depth, dropout, high dimensionality, batch effects, and the fact that mRNA abundance does not necessarily predict protein abundance or function (87–95). Proteomics can identify circulating or tissue-level protein biomarkers, but serum proteomics is challenged by abundant proteins, dynamic range, post-translational modifications, and platform-dependent detection (5, 96–98). Metabolomics and lipidomics are closer to phenotype but are sensitive to diet, fasting, collection time, medications, extraction method, ionization efficiency, and annotation uncertainty (99–104). WES adds genetic susceptibility information but misses non-coding regulatory variants and cannot by itself distinguish causation from association (105–108).

Bioinformatic tools should therefore be selected according to the biological question rather than listed as interchangeable methods. Differential expression tools such as DESeq2, edgeR, limma-voom, or microarray-specific limma pipelines are appropriate for identifying genes or transcripts that vary between conditions when study design, normalization, and multiple-testing correction are specified (109–113). GO, KEGG, and GSEA translate gene-level changes into pathways, but pathway databases are incomplete and can overrepresent broad inflammatory processes (114–118). Cell communication tools such as CellChat, NicheNet, or ligand–receptor analyses can generate hypotheses about intercellular signaling, but they infer potential communication from expression rather than proving signaling activity (119, 120). WGCNA and high-dimensional WGCNA identify correlated gene modules (121, 122), whereas LASSO, SVM-RFE, random forests, and other machine-learning methods can nominate predictive features (123, 124). These approaches require feature selection independent validation, careful cross-validation, and external cohorts to avoid information leakage and overfitting, especially in the small datasets common in LE research (125).

### 7.3 Limitations of the current literature

The current evidence base remains limited by small sample sizes, frequent single-center designs, heterogeneous controls, and variable validation strategies. Controls range from contralateral unaffected tissue to unrelated healthy donors, surgical controls, or disease-risk controls, making cross-study comparisons difficult. Cross-study integration is further limited by inconsistent preprocessing pipelines, incomplete clinical and technical metadata, variable endpoint definitions, and limited availability of analysis code and reusable processed data. Many studies are cross-sectional, so they cannot distinguish initiating events from late tissue remodeling. Mouse, sheep, and *in vitro* models permit mechanistic testing but may not fully reproduce human cancer treatment, obesity, infection, age, sex, and limb-specific biology. A further limitation is that most available studies focus on BCRL, whereas lymphatic filariasis is the most common cause of secondary LE worldwide. Omics at the parasite–human host interface remains markedly understudied (126).

### 7.4 Future directions and emerging technologies

Future studies should prioritize prospective, multi-center cohorts with standardized clinical phenotyping, sample timing, tissue site, processing protocols, and control definitions. Longitudinal sampling before lymphatic injury, during subclinical swelling, and after interventions such as compression, lymphaticovenous anastomosis, vascularized lymph node transfer, immunomodulation, or anti-fibroadipose therapy would help define causal biomarkers and treatment-response signatures. Spatial transcriptomics, spatial proteomics, multiplexed imaging, epigenomics, ATAC-seq, DNA methylation profiling, WES, whole-genome sequencing, and integrated multi-omics should be combined with functional assays of lymphatic pumping, permeability, immune infiltration, fibrosis, and adipose expansion.

Several outstanding questions should guide the next phase of the field. Which omics signatures distinguish patients destined to progress from those with stable or reversible swelling? Are CD4^+^ T cells, Tregs, macrophages, adipose progenitors, fibroblasts, and LMCs drivers of disease or secondary responders at different stages? Can therapies restore lymphatic barrier function and contractility after injury, or must they also suppress fibroadipose and immune memory programs? Which biomarkers are specific to LE rather than general chronic inflammation or obesity? How do inherited variants interact with surgery, radiation, infection, chemotherapy, immunotherapy, age, and BMI to determine risk?

Artificial intelligence, machine learning, large language models, and AI-assisted drug discovery (AIDD) may accelerate this work if they are anchored in validated biology. Omics-informed models can prioritize drug targets, predict patient subgroups, identify repurposing opportunities, and simulate perturbation of cell–cell signaling networks such as CXCL12–CXCR4, TIE2, PPARγ, TRPV4, IGF1R, and 15-lipoxygenase pathways. AIDD could be particularly useful in secondary LE because no approved pharmacologic cure exists and candidate mechanisms span immune, fibrotic, adipose, vascular, and metabolic biology (127, 128). However, these approaches will require transparent feature selection, independent validation, mechanistic follow-up, and clinically meaningful endpoints before they can guide therapy.

### 7.5 Conclusion

Omics studies have begun to redefine secondary LE as a multicellular, inflammatory, fibroadipose, vascular, metabolic, and genetically modulated disease process. The field now has candidate pathways and biomarkers with plausible diagnostic and therapeutic relevance, but most findings remain hypothesis-generating. Progress will depend on larger and more diverse cohorts, standardized methodology, cell- and spatially resolved measurements, rigorous bioinformatic validation, and functional experiments that connect molecular signatures to lymphatic transport, tissue remodeling, and patient outcomes.
